# Supplementary figures and images for: Application of Novel Transcription Factor Machine Learning Model and Targeted Drug Combination Therapy Strategy in Triple Negative Breast Cancer
Source: Int J Mol Sci. 2023 Aug 31;24(17):13497. doi: 10.3390/ijms241713497 (PMC10487460; doi:10.3390/ijms241713497)

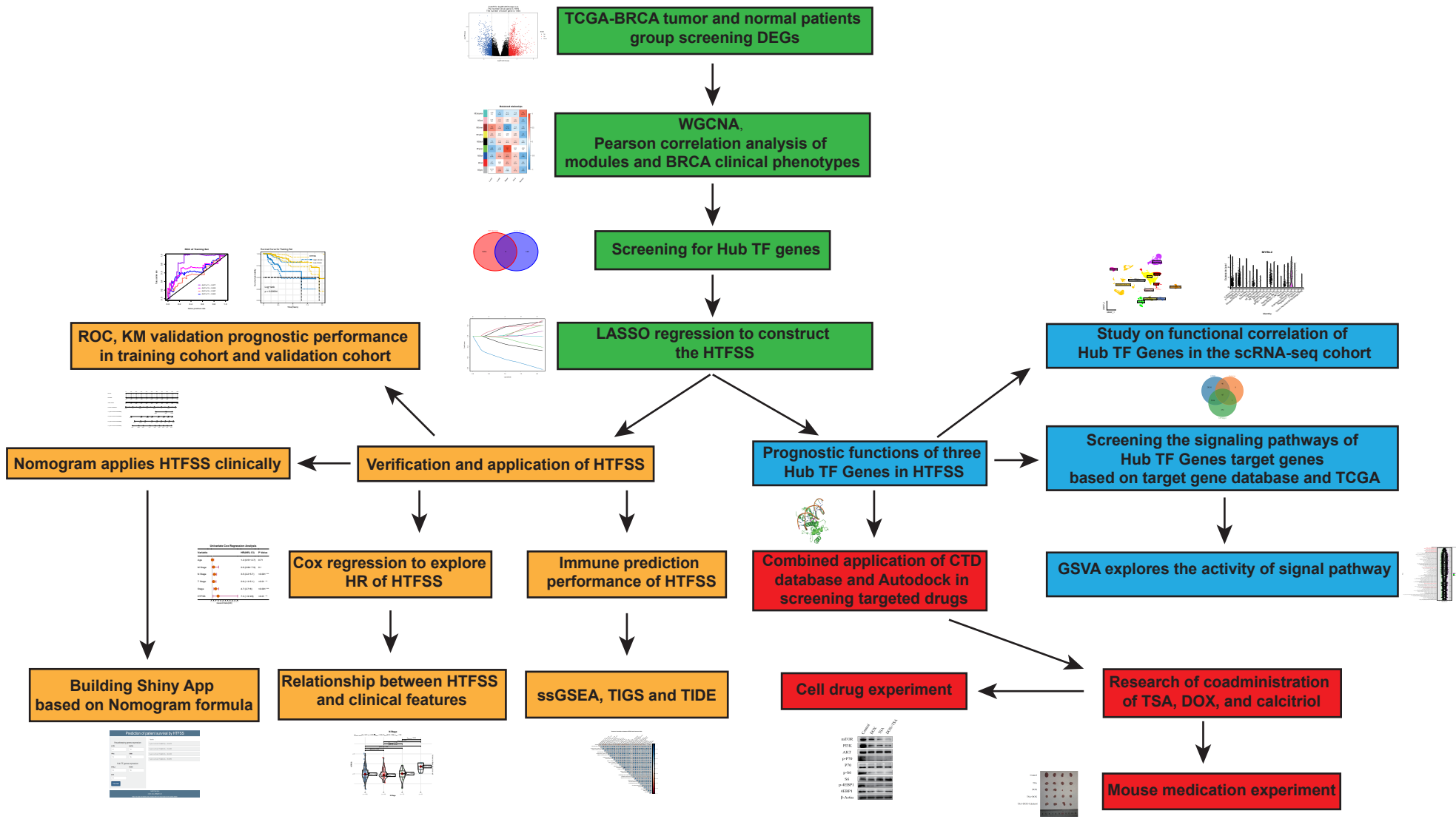

Supplement: Supplementary file 1 [file ijms-24-13497-s001.zip › Figure S1.pdf]

A

Enrichment: KEGG pathway

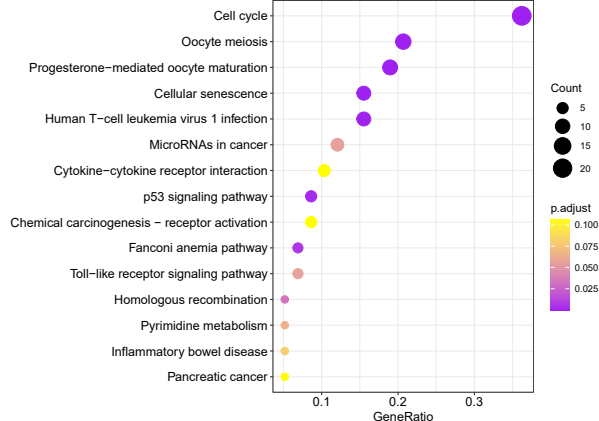

B

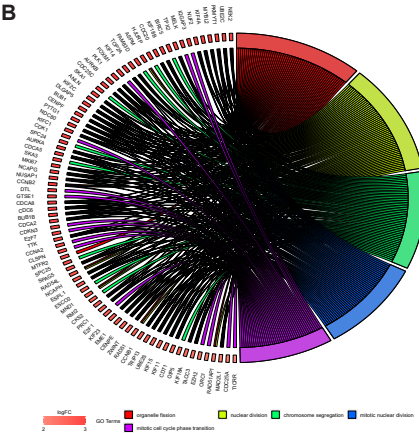

C

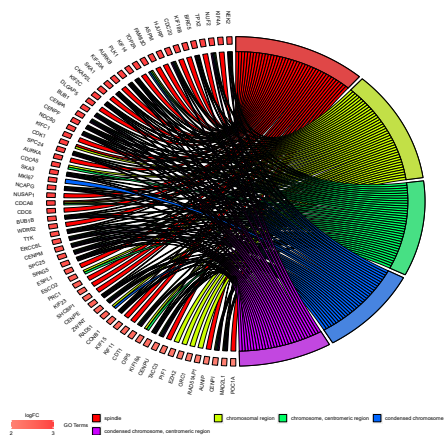

D

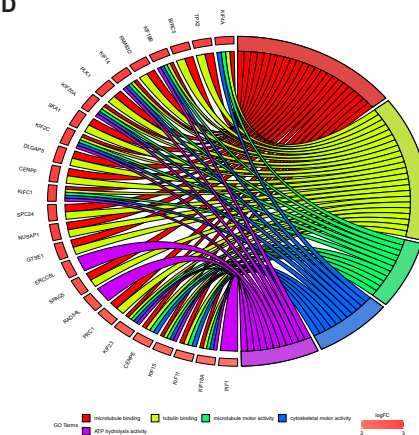

Supplement: Supplementary file 1 [file ijms-24-13497-s001.zip › Figure S2.pdf]

**A**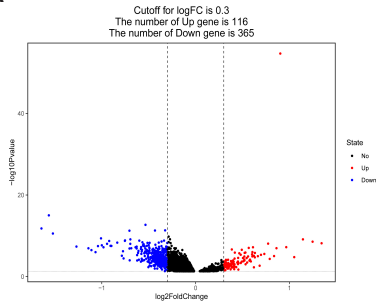**B**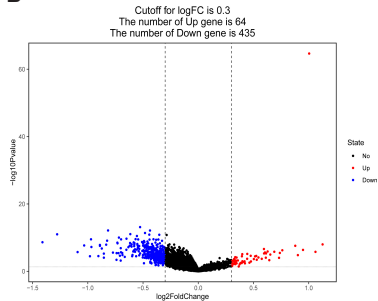**C**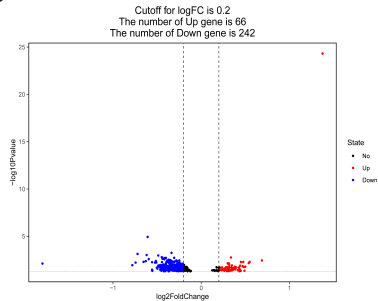**D**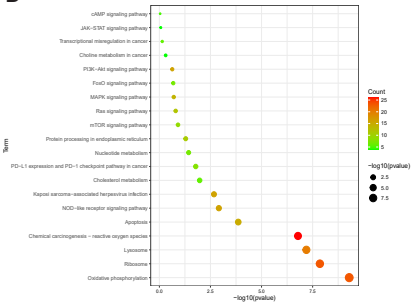**E**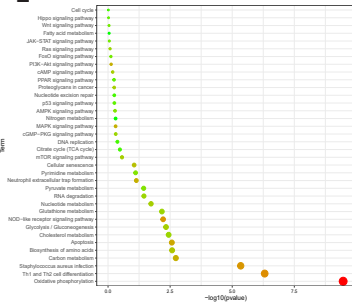**F**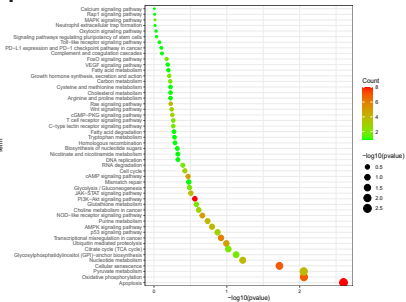

Supplement: Supplementary file 1 [file ijms-24-13497-s001.zip › Figure S3.pdf]

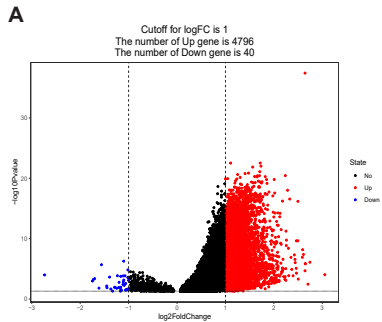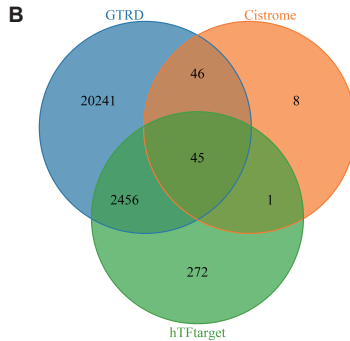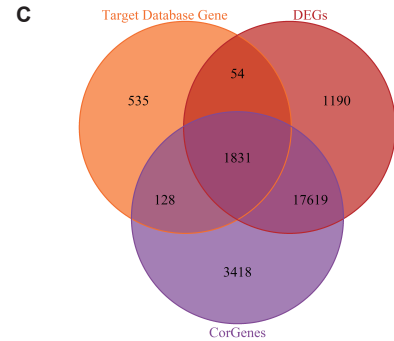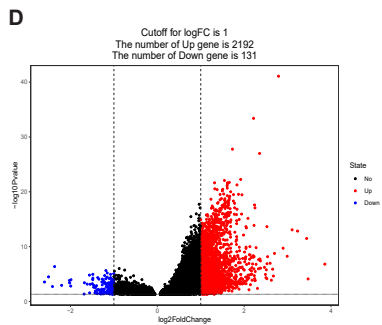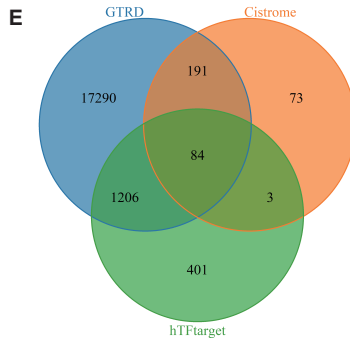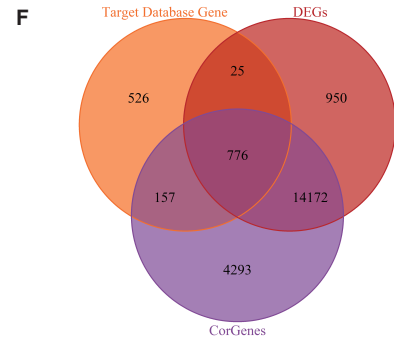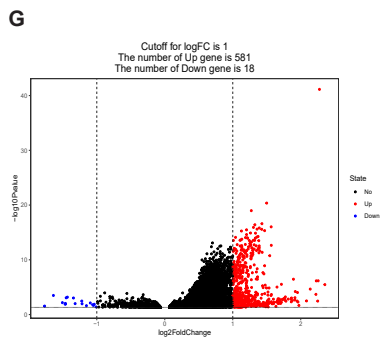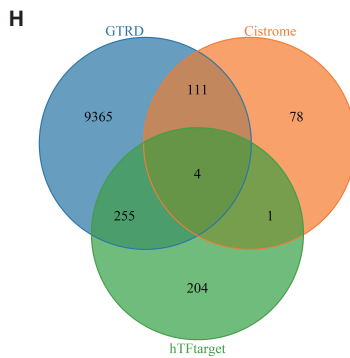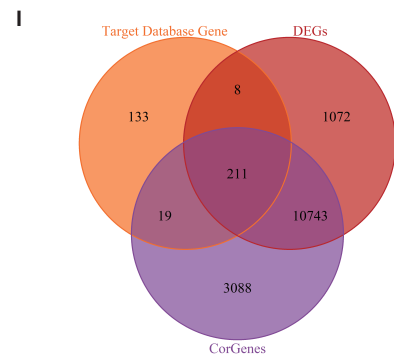

Supplement: Supplementary file 1 [file ijms-24-13497-s001.zip › Figure S4.pdf]
